# Faithful AI in Medicine: A Systematic Review with Large Language Models and Beyond

**DOI:** 10.21203/rs.3.rs-3661764/v1

**Published:** 2023-12-04

**Authors:** Qianqian Xie, Edward J. Schenck, He S. Yang, Yong Chen, Yifan Peng, Fei Wang

**Affiliations:** Weill Cornell Medicine; New York-Presbyterian Hospital, Weill Cornell Medical Center; Weill Cornell Medical College; University of Pennsylvania; Weill Cornell Medicine; Weill Cornell Medicine

**Keywords:** large language models, factuality and reliability, generative medical AI, evaluation metrics

## Abstract

**Objective:**

While artificial intelligence (AI), particularly large language models (LLMs), offers significant potential for medicine, it raises critical concerns due to the possibility of generating factually incorrect information, leading to potential long-term risks and ethical issues. This review aims to provide a comprehensive overview of the faithfulness problem in existing research on AI in healthcare and medicine, with a focus on the analysis of the causes of unfaithful results, evaluation metrics, and mitigation methods.

**Materials and Methods:**

Using PRISMA methodology, we sourced 5,061 records from five databases (PubMed, Scopus, IEEE Xplore, ACM Digital Library, Google Scholar) published between January 2018 to March 2023. We removed duplicates and screened records based on exclusion criteria.

**Results:**

With 40 leaving articles, we conducted a systematic review of recent developments aimed at optimizing and evaluating factuality across a variety of generative medical AI approaches. These include knowledge-grounded LLMs, text-to-text generation, multimodality-to-text generation, and automatic medical fact-checking tasks.

**Discussion:**

Current research investigating the factuality problem in medical AI is in its early stages. There are significant challenges related to data resources, backbone models, mitigation methods, and evaluation metrics. Promising opportunities exist for novel faithful medical AI research involving the adaptation of LLMs and prompt engineering.

**Conclusion:**

This comprehensive review highlights the need for further research to address the issues of reliability and factuality in medical AI, serving as both a reference and inspiration for future research into the safe, ethical use of AI in medicine and healthcare.

## Introduction

**Figure 1:**
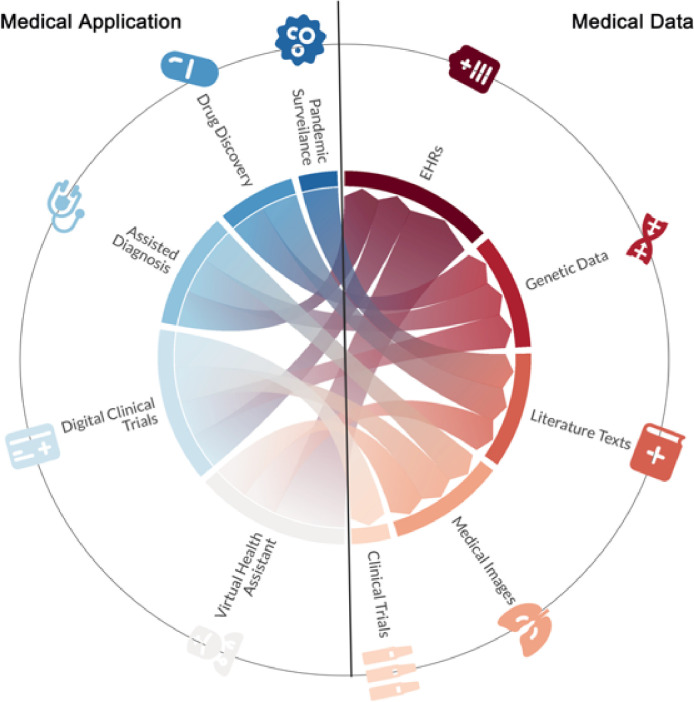
The example of medical data and realistic medical applications with AI.

Artificial intelligence (AI) has been gradually applied in different aspects of healthcare and medicine^1–3^. Its benefits are seen in various fields such as quickening medical research, assisting in the detection and diagnosis of diseases, providing tailored health recommendations, and much more (Fig. 1). The success of Medical AI has been closely tied to the development of fundamental AI algorithms [4] and the availability of various biomedical data, including abundant unlabeled data and labeled data annotated by experts.

Recently, pre-trained language models (PLMs) [5] such as BERT [6], which are pre-trained on a huge amount of unlabeled language texts in a self-supervised learning manner (typically based on the new backbone neural network architecture called Transformer [7] ), have revolutionized natural language processing (NLP). They demonstrated strong potential in Medical AI [5], including tasks beyond NLP like pathology detection [8, 9]. More recently, large language models (LLMs) [10], exemplified by ChatGPT [11] and GPT-4 [12] developed by OpenAI, have exhibited impressive capabilities to understand complex natural language input and produce human-like text content. LLMs, with their significantly larger model and pre-training data sizes, exhibit remarkably stronger generalizability than PLMs [13], and unlocked new abilities such as complex reasoning and in-context learning; namely LLMs can perform new tasks without task-specific training but seeing a few examples with task-specific natural language explanation texts [10]. For example, GPT-4 has been reported to pass the threshold of the United States Medical Licensing Exam (USMLE) and showed great potential in assisting clinical decision-making [12, 14]. These recent advancements represent a potential inflection point for medical AI in several ways: 1) remarkable generalization ability and in-context learning ability, allowing them to be applied to diverse medical tasks without task-specific training, 2) user-friendly design, accepting natural language inputs, enabling easy access for laypeople and medical professionals, 3) fast growing, constantly push forward the development of medical AI.

However, a key concern is their potential risk of generating non-factual or unfaithful information, commonly referred as the faithfulness problem [15]. Specifically, generative AI methods can generate content that is factually inaccurate or biased. The faithfulness problem can pose long-term risks such as preventing medical innovation due to providing wrong guidance in medical research and misallocating medical resources due to providing unfaithful information on disease treatment. Moreover, ethical issues can be caused by the problem, such as deteriorating trust in healthcare by providing misleading medical information to patients and practitioners. This could lead to unintended consequences, such as misleading clinical decisions and a negative impact on patient care.

## What is the Faithfulness Problem?

### Definition and Categorization

Generative medical AI systems^16–18^ learn to map from various types of medical data such as EHRs, medical images or protein sequences, to desired output such as the summarization or explanation of medical scans and three-dimensional (3D) protein structures. A medical AI system is considered unfaithful or to have a factual inconsistency issue, also known as “hallucination” in some studies^19,20^, if it generates content that is not supported by existing knowledge, reference, or data. The factual inconsistency issue in generative medical AI generally can also be categorized into the following two major types:

- Intrinsic Error: the generated output contradicts existing knowledge, reference, or data. For example, if there is a sentence in the radiology report summary generated by the AI system “The left-sided pleural effusion has increased in size”, but the actual sentence in the radiology report is “There is no left pleural effusion”. Then the summary contradicts facts contained in the input data.
- Extrinsic Error: the generated output cannot be confirmed (either supported or contradicted) by existing knowledge, reference, or data. For example, if the content of the radiology report includes: “There is associated right basilar atelectasis/scarring, also stable. Healed right rib fractures are noted. Linear opacities projecting over the lower lobe are also compatible with scarring, unchanged. There is no left pleural effusion”, and the generated summary includes: “Large right pleural effusion is unchanged in size”. Then this piece of information cannot be verified by the given radiology report, since the information about “right pleural effusion” is not mentioned there.

Different medical tasks utilize varied reference standards to evaluate the accuracy and faithfulness of AI-generated content. For instance: In a text summarization system, where the goal is to condense medical documents like study protocols, biomedical literature, or clinical notes, the primary reference is the original document itself. This is to ensure that the summarized content remains true to the source. To offer a clearer perspective, Table 1 summarizes the reference standards used in various medical AI tasks to evaluate the factual consistency of AI-generated outputs.

## Why is there a Faithfulness Problem?

The factual inconsistency of medical AI systems can be attributed to a wide range of reasons.

### Limitation of backbone language model

PLMs and the latest LLMs, commonly used for representing and processing diverse medical data, have limitations on remembering and recognizing medical facts. Several factors contribute to this: 1) Most PLMs and LLMs weren’t initially trained with an extensive amount of medical data, making their understanding of the domain limited. 2) Smaller Scale of Biomedical Training Data: While there are medical-focused PLMs like BioBERT^21^and BlueBERT^22^, they’re trained on smaller datasets compared to general-purpose PLMs and LLMs. 3) Privacy Restrictions: Essential biomedical records, such as Electronic Health Records (EHRs) or radiology reports, are often inaccessible for training due to privacy concerns. As a result, existing domain-specific PLMs were mainly pre-trained with biomedical literature texts, that are easy to access on a large scale. While abundant, it might not always cover the day-to-day realities of medicine.

### Data discrepancy

The factual inconsistency problem also arises from the discrepancies between the “ground truth” (the desired model output used for training) and the reference standards (the standard used for evaluating the factuality of generated outputs) as shown in Table 1 on some medical generation tasks^19,20^. For example, in the task of text summarization, the ‘ground truth’ consists of summaries of biomedical articles crafted by domain experts. Models are trained to produce summaries that mirror these expert-written ground truths. When evaluating the model’s outputs for accuracy and factuality, the reference used is the comprehensive original content of the biomedical articles. However, a challenge emerges: while the expert-written summaries aim to encapsulate the main points, they might occasionally omit certain key aspects from the original articles. Consequently, even if a model’s output aligns perfectly with the ground truth, it may inadvertently miss details crucial to the reference. This isn’t necessarily a flaw in the model’s learning but highlights the potential for the discrepancy between the ground truth and the reference.

### Limitations of decoding

Another cause for the factual inconsistency problem is the limitation of medical AI systems’ decoding strategy, namely the way they generate information. One notable issue is the “exposure bias” problem^23,24^. When training these systems, they learn to create the next piece of information (referred to as a ‘token’, which is like a word or phrase with a specific meaning) by looking at previous correct examples. However, in real-world situations, these AI systems can’t always see the correct examples. They must make predictions based on their own past responses. Many of the current methods prioritize generating a smooth flow of text over ensuring that every fact they produce is accurate, which might increase the chances of producing incorrect information.

### Objective

In this review, we provide an overview of the research on the faithfulness problem in existing medical AI studies, including cause analysis, evaluation metrics, and mitigation methods. We comprehensively summarize the recent progress in maintaining factual correctness in various generative medical AI models, including knowledge-grounded large-language models, text-to-text generation tasks such as medical text summarization and simplification, multimodality-to-text generation tasks such as radiology report generation, and automatic medical fact-checking. We discuss the challenges of maintaining the faithfulness of AI-generated information in the medical domains, as well as the forthcoming opportunities for developing faithful medical AI methods. The goal of this review is to provide researchers and practitioners with a blueprint of the research progress on the faithfulness problem in medical AI, help them understand the importance and challenges of the faithfulness problem, and offer them guidance for using AI methods in medical practice and future research.

## Methods

### Search Strategy

We conducted a comprehensive search for articles published between January 2018 to March 2023 from multiple databases, including PubMed, Scopus, IEEE Xplore, ACM Digital Library, and Google Scholar. We used two groups of search queries: 1) faithful biomedical language models: factuality/faithfulness/hallucination, biomedical/medical/clinical language models, biomedical/medical/clinical knowledge, 2) mitigation methods and evaluation metrics: factuality/faithfulness/hallucination, evaluation metrics, biomedical/medical/clinical summarization, biomedical/medical/clinical text simplification; radiology report summarization, radiology report generation, medical fact-checking.

### Filtering Strategy

**Figure 2:**
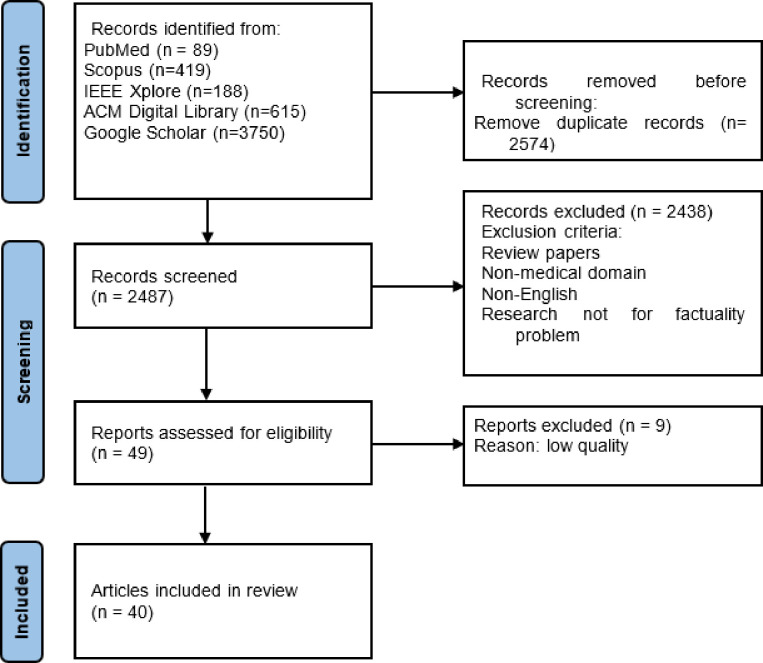
The process of article selection.

A total of 5,061 records were sourced from five databases, with 2487 articles remaining post-duplicate removal. These were screened based on title, abstract, and exclusion criteria such as being a review paper, non-English content, irrelevance to the medical domain, or the factuality issue, leaving 49 articles (see Fig. 2 for the full process of article selection). Two independent reviewers (the first author and a fellow Ph.D. candidate with expertise in NLP and bioinformatics) screened these articles. Before initiating the screening, both reviewers collaboratively defined and standardized the exclusion criteria to ensure an objective and consistent evaluation process. Areas of disagreement between the two reviewers were resolved through discussion until a consensus was reached. If an agreement could not be achieved, a third reviewer was consulted (a postdoc with expertise in NLP and bioinformatics). After a full-text review, 9 were further excluded due to low content quality per the GRADE criteria^77^. The same two reviewers further adapted GRADE to evaluate individual research articles for quality and relevance. The assessment using GRADE revolved around four main criteria: 1) Risk of Bias: Each article was evaluated for potential biases, including selection bias, performance bias, detection bias, attrition bias, and reporting bias. Articles that exhibited high risks in multiple domains were flagged for potential exclusion. 2) Inconsistency: We checked for any unexplained heterogeneity or variability in study results. Inconsistencies in the reported results or methodologies without adequate explanations led to a downgrade in quality. 3) Indirectness: Articles that didn’t directly address the objectives of our review or that had populations, interventions, or outcomes that varied significantly from our scope were considered less direct. 4) Imprecision: Studies with wide confidence intervals, sparse data, or that didn’t provide sufficient detail to assess the reliability of their results were flagged for potential exclusion. For each article, the reviewers assigned a quality grade of high, moderate, low, or very low based on the accumulation of these criteria. Articles with a ‘very low’ quality grade, indicating high levels of bias, inconsistency, indirectness, or imprecision, were excluded from the review.

## Results

To improve the faithfulness of LLMs, many efforts have been focused on improving the backbone model with medical knowledge and optimizing the factual correctness of AI medical systems.

### Faithfulness in Large Language Models (LLMs)

Some efforts (see Table 2) have focused on explicitly incorporating extra medical knowledge to address the factuality problem in domain-specific PLMs and LLMs. Yuan et al^79^ proposed to train a knowledge-aware language model by infusing entity representations, as well as entity detection and entity linking pre-training tasks based on the Unified Medical Language System (UMLS) knowledge base. The proposed method improves the performance of a series of biomedical language models, such as BioBERT and PubMedBERT, on the named entity recognition (NER) and relation extraction (RE) tasks. Jha et al^80^ proposed to prob diverse medical knowledge bases into language models with the continual knowledge infusion mechanism to avoid forgetting encoded knowledge previously when injecting multiple knowledge bases. This improves several biomedical language models, such as BioBERT and PubMedBERT, in medical question answering, named entity recognition and relation extraction. Singhal et al^25^ proposed the Med-PaLM that aligns a 540-billion parameter LLM PaLM^26^ into the medical domains by instruction prompt tuning, which greatly alleviates errors of PaLM on scientific grounding, harm, and bias from low-quality feedback. Zakka et al^27^ proposed the knowledge-grounded LLMs Almanac, which retrieves information from medical databases for replying to clinical queries, rather than directly generating content with LLMs. Almanac has shown better performance than ChatGPT on medical Q&A based on the 20 questions derived from their ClinicalQA dataset and mitigates the factual inconsistency problem. Nori et al^28^ conducted a comprehensive study on the ability of GPT-4 on medical competency examinations and medical Q&A benchmarks recently, where GPT-4 shows much better performance than GPT-3.5. Although their assessment highlights the great potential of GPT-4 in assisting healthcare professionals, they suggested GPT-4 still has a large gap in safe adoption in the medical domain like prior LLMs, due to several limitations such as the risk of error generations, biases, and societal issues.

### Faithfulness of AI Models in Different Medical Tasks

Many efforts (see Table 3) have been devoted to optimizing the factuality of generative methods in medicine and healthcare, as well as their factuality evaluation for a specific task with various techniques such as incorporating medical knowledge, reinforcement learning, and prompt learning.

### Medical Text Summarization

Medical text summarization^29–33^ is an important generative medical task, with the goal of condensing medical texts such as scientific articles, clinical notes, or radiology reports into short summaries.

#### Evaluation Metrics

Existing commonly used evaluation metrics in text summarization, such as ROUGE^36^ and BERTScore^45^, have been proven to be ineffective in evaluating factual correctness, especially in the medical domain. For RRS, Zhang et al^17^ proposed the CheXpert F1 score that calculates the overlap of 14 clinical observations, such as “enlarged cardiom” and “cardiomegaly”, between the generated summary and the reference summary. Delbrouck et al^35^ further proposed a RadGraph score that calculates the overlap of medical entities and relations^46^ between the generated summary and the gold summary and can be used for various modalities and anatomies. For the biomedical literature summarization, Wallace et al^40^ proposed findings-Jensen-Shannon Distance (JSD) calculating the agreement of evidence directions of the generated summary and reference summary of the systematic review, according to JSD. Based on findings-JSD, Deyoung et al^38^ further proposed the improved metric DEI that calculates the agreement of the intervention, outcome, and evidence direction based on the Jensen-Shannon Distance, between the generated summary and the input medical studies. Otmakhova et al^47^ proposed the human evaluation approach for medical study summarization, where they defined several quality dimensions, including PICO correctness, evidence direction correctness, and modality to evaluate the factuality of the generated summary. Based on the human evaluation protocol, Otmakhova et al^48^ further developed the Dloss to evaluate the factual correctness of the generated summary on different aspects such as strong claim, no evidence, no claim, etc., and evidence directions. Adams et al^49^ did a meta-evaluation on existing automatic evaluation metrics, including BARTScore, BERTScore, CTC^50^ and SummaC^51^ on assessing long-form hospital-course summarization. They found that automatic evaluation metrics correlate well with human annotations in the biomedical domain but struggle to assess factual errors needing deep clinical knowledge, like missingness and incorrectness.

#### Optimization Methods

The factual inconsistency problem was first explored in the radiology report summarization (RRS). Specifically, Zhang et al^34^ found that nearly 30% of radiology report summaries generated from the neural sequence-to-sequence models contained factual errors. To deal with the problem, Zhang et al^17^ proposed to optimize the factual correctness of the radiology report summarization methods with reinforcement learning (RL). Their method with RL could improve the factuality evaluation metric CheXpert F1 score by 10% when compared with the baseline method. Delbrouck et al^35^ designed a summarization method that optimized the RadGraph score (a factuality evaluation metric) reward with RL. Their method could consistently improve the factual correctness and quality of the generated summary, where the RadGraph score, CheXpert F1, ROUGE-L^36^ are improved by 2.28%−4.96%, 3.61%−5.1%, and 0.28%−0.5%. Xie et al^37^ proposed the two-stage summarization method FactReranker, which aims to select the best summary from all candidates based on their factual correctness scores by incorporating the medical factual knowledge based on the RadGraph. FactReranker achieves the new SOTA on the MIMIC-CXR dataset and improves the RadGraph score, F1CheXbert, and ROUGE-L by 4.84%, 4.75%, and 1.5%.

For medical studies, Deyoung et al^38^ found that while BART-based summarizers^39^ could create fluent summaries for medical studies, maintaining faithfulness was a challenge. For instance, only 54% of the summaries agreed on the intervention’s effect direction as compared to the original systematic review. Wallace et al^40^ proposed the decoration and sorting strategy that explicitly informed the model of the position of inputs conveying key findings, to improve the factual correctness of the generated summary from the BART-based summarizer for published reports of randomized controlled trials (RCTs). Alambo^41^ studied the factual inconsistency problem of a transformer-based encoder decoder summarization method. They proposed to integrate the biomedical named entities detected in input articles and medical facts retrieved from the biomedical knowledge base to improve the model’s faithfulness. Yadav et al^42^ proposed to improve the factual correctness of the generated summary for medical questions, by maximizing the question type identification reward and question focus recognition reward with the policy gradient approach. Chintagunta et al^43^ investigated using GPT-3 to generate higher-quality labeled data, which has proven to be able to train the summarizer with better factual correctness. Liu et al^44^ proposed the task of automatically generating discharge instructions based on the patients’ electronic health records and the Re3Writer method for the task, which retrieved related information from discharge instructions of previous patients and medical knowledge to generate faithful patient instructions.

### Medical Text Simplification

Medical text simplification^33^ aims to simplify highly technical medical texts to plain texts that are easier to understand by non-experts such as patients. It can greatly improve the accessibility of medical information.

#### Evaluation Metrics

Similar to the medical text summarization, automatic similarity-based evaluation metrics such as ROUGE were also used for evaluating the semantic similarity between outputs and references in the medical text simplification. Other crucial aspects include readability and simplicity, evaluated using metrics like the Flesch-Kincaid grade level (FKGL)^56^. Intuitively, the PLMs pre-trained on the technical corpus can assign higher likelihoods to the technical terms than that pretrained on the general corpus. Based on this intuition, Devaraj et al^53^ proposed a new readability evaluation metric calculating the likelihood scores of input texts with a masked language model trained on the technical corpus. Devaraj et al^57^ proposed a RoBERTa-based method to classify factual errors in text simplification, like insertions, deletions, and substitutions.

#### Optimization Methods

Lu et al^52^ proposed the summarize-then-simplify method for paragraph-level medical text simplification, that uses narrative prompts with key phrases to encourage the factual consistency between the input and the output. Their proposed method significantly outperforms the BART-based simplification method^53^ by 0.49 on the 5-point scale on the factuality of outputs. Jeblick et al^54^ used ChatGPT to simplify 45 radiology reports and had them evaluated by 15 radiologists. The radiologists mostly found the outputs factually correct, but noted errors such as misinterpretation of medical terms, imprecise language, and other factual mistakes. Lyu et al^55^ also evaluated the performance of ChatGPT on simplifying radiology reports to plain language on 62 chest CT screening reports and 76 brain MRI screening reports. ChatGPT showed good performance, scoring 4.268 on a 5-point scale by radiologists, with an average of 0.08 and 0.07 instances of missing and inaccurate information respectively.

### Radiology Report Generation

Radiology report generation aims to automatically generate radiology reports illustrating clinical observations and findings with the input medical images such as chest X-rays and MRI scans. It can help to reduce the workload of radiologists and improve the quality of healthcare.

#### Evaluation Metrics

To evaluate the clinical correctness of the generated radiology reports, some efforts^58,59,62^ proposed to use of the CheXpert-based metric to evaluate the overlap of 14 clinical observations between generated reports and references annotated by the CheXpert. Delbrouck et al^60^ proposed the RadGraph score to calculate the overlap of the clinical entities and relations between generated reports and references annotated by the RadGraph. Recently, Yu et al^63^ examined the correlation between existing automatic evaluation metrics including BLEU^64^, BERTScore, F1 CheXpert, and RadGraph F1, and the score given by radiologists on evaluating the factuality of the generated reports. They found that the evaluation results of F1 CheXpert and BLEU were not aligned with those of radiologists, and BERTScore and RadGraph F1 were more reliable. They further proposed a new evaluation metric RadCliQ, which is the weighted sum of the score from BLEU and RadGraph F1 based on their optimized coefficients. It showed better alignment with the evaluation of radiologists than the above four metrics.

#### Optimization Methods

Most existing efforts adopted RL to optimize the factual correctness of radiology report generation methods. Nishino et al^58^ proposed an RL method, optimizing a clinical reconstruction score for improved factual correctness in reports. This method led to a 5.4% improvement in the CheXpert factuality metric’s F1 score compared to the model without RL optimization. Miura^59^ proposed an RL method to optimize the entity match score between generated and reference reports, improving consistency. This method significantly improved the CheXpert F1 score by 22.1% compared to baselines. Delbrouck et al^60^ designed the RadGraph reward calculating the overlap of entities and relations between the generated report and the reference, based on the RadGraph dataset including annotated entities and relations of the Chest X-ray reports. The proposed method improves the factuality evaluation metric F1 RadGraph score by 5.5% on the MIMIC-CXR dataset when compared with baselines^59^. Nishino et al^61^ further proposed an RL-based method Coordinated Planning (CoPlan) with the fact-based and description-order-based evaluator, to encourage the model to generate radiology reports that are factually and chronologically consistent with reference reports. Their method outperforms the baseline T5 model on clinical factual accuracy by 9.1%.

### Medical Fact Checking

Automatic medical fact-checking, which verifies the truthfulness of claims in a medical text, is a promising tool for detecting and correcting factual errors in medical generative methods. Many existing efforts have contributed to the creation of medical fact-checking data resources^65–72^, such as PUBHEALTH^65^ and HEALTHVER^68^ et al. Kotonya et al^65^ proposed an explainable automatic fact-checking method using a classifier based on pre-trained language models like BERT, SciBERT^73^, BioBERT, and a BERTSum-based summarization model for generating explanations. On the PUBHEALTH dataset, the SciBERT-based method achieved the highest macro F1, precision, and accuracy scores. Wadden et al^66^ proposed an automatic fact-checking pipeline that retrieves abstracts based on input claims using TD-IDF similarity, selects rationale sentences, and predicts the labels of abstracts relative to the claims using BERT-based models. Among several sentence encoders like SciBERT, BioMedRoBERTa, and RoBERTa-base, RoBERTa-large showed the best performance in label prediction. Wadden et al^74^ proposed MULTIVERS for predicting fact-checking labels for a claim and corresponding evidence abstract. It uses the Long-former^75^ encoder to process the long sequences and employs multi-task learning to align abstract-level labels with sentence-level ones. MULTIVERS outperformed existing methods in zero-shot and few-shot settings on three medical fact-checking datasets.

## Discussion

With all the reviews of existing works on faithful AI in healthcare and medicine above, we will discuss the overall limitations of existing studies and future research directions.

### Datasets

#### Unlabeled Data for Self-supervised Learning

PLMs and LLMs trained with self-supervised learning, requiring large-scale unlabeled medical data. However, collecting such data is difficult due to privacy and cost considerations. For example, the unlabeled clinical data used to train the clinical PLMs such as ClinicalBERT^76^ is 3.7GB, while that used to train LLMs such as GPT-4 can be up to 45TB. Moreover, most datasets are limited to a single language, where English is predominantly used, and a single data modality. This can hinder the development of faithful medical AI methods in low-resource and rural areas. Therefore, it’s crucial to develop multimodal, multilingual PLMs to improve the generalization and faithfulness of medical language models, using various languages and data types, such as text and images.

#### Annotated Data for Supervised Learning and Evaluation

Developing and evaluating faithful medical AI methods relies on high-quality annotated medical data. Collecting large-scale high-quality annotated medical data is even more challenging, due to the high cost of both time and expertise. Existing annotated datasets are usually small, with no publicly accessible data for the meta-evaluation of automated metrics in most medical tasks. This makes it difficult to verify the reliability of these metrics. Thus, building domain expert annotated datasets for various medical tasks is essential to analyze metric alignment with expert preferences and develop reliable automatic metrics and effective mitigation methods. methods.

### Backbone Models

#### Biomedical Domain-Specific Language Models

Many existing medical AI methods use biomedical PLMs and fine-tune them with task-specific datasets for various downstream tasks. However, these models, typically pre-trained with the biomedical literature texts and a few other types of medical texts, capture limited medical knowledge. Moreover, their sizes are typically small (usually less than 1B parameters). For example, up to now, the largest PLMs in the medical domain, PubMedGPT, has 2.7B parameters, which is far smaller than the scale of LLMs in the general domain (e.g., GPT-3.5 with 175B parameters). Therefore, to improve the reliability of biomedical PLMs, future strategies could include larger model sizes and multimodal data training.

#### Large Generative Language Models

LLMs have shown amazing natural language understanding and generation abilities. However, they are not mainly trained with data in the medical domain, and none are publicly available, hindering the development of reliable medical AI. Therefore, adapting LLMs for the medical field is vital, with strategies like fine-tuning with domain-specific data and prompt tuning with human feedback. There is a recent work^25^ that aligns the LLM PaLM with the medical domain. Unfortunately, it is still not publicly available.

### Faithful Medical AI Methodologies

#### Mitigation Methods

Although factuality is a critical issue in existing medical AI methods, little effort has been devoted to improving the faithfulness of backbone language models and medical AI methods for downstream tasks. No research has investigated the factuality in medical tasks, including medical dialogue generation, medical question answering, and drug discovery et al. It is also important to develop explainable medical AI methods, especially LLMs. The explanation can play an important role in alleviating the hallucination problem. It helps to understand and trace the causes of factual errors, makes it easier to assess factual errors, and enhances medical AI faithfulness.

#### Incorporating Medical Knowledge

Existing efforts have proven the effectiveness and importance of improving the factuality in both backbone language models and medical AI methods for specific tasks by incorporating medical knowledge. However, most focused on extracting medical knowledge from external biomedical knowledge bases. Recently, there has been an effort21 investigating the efficiency of instruction prompt tuning on injecting medical knowledge into LLMs, which relies on human feedback and thus can be expensive and time-consuming. Effective incorporation of medical knowledge in efficient and scalable ways remains a critical challenge.

### Evaluations

#### Automatic Evaluation Metrics

Existing automatic evaluation metrics, calculating the overlap of medical facts between outputs generated by the algorithm and references, fail to distinguish different types of factual errors, such as intrinsic and extrinsic errors, as introduced in Section 3.1. In addition, the assessment of factuality relies on human evaluations for many tasks, such as medical question answering, without automatic factuality evaluation metrics. Future work should explore fine-grained automatic metrics to clarify and assess varied medical factual errors and a unified evaluation guideline for standardized criteria across medical tasks.

#### Meta-evaluation

Assessing the effectiveness of automatic evaluation metrics is critical for correctly evaluating the factuality of methods. Otherwise, the ineffective automatic evaluation metrics can misguide the optimization and evaluation of methods. There is rare work^63^ investigating the meta-evaluation of automatic factuality metrics used in various medical tasks and analyzing their alignment with domain experts. Therefore, it is important to conduct the prospective randomized controlled trial (RCT) level assessment for these evaluation metrics to evaluate their reliability and effectiveness.

## Conclusion

The progress of fundamental AI methods, especially the most recent LLMs, provides great opportunities for medical AI, but there is a severe concern about the reliability, safety, and factuality of generated content by medical AI methods. In this review, we provide the first comprehensive overview of the faithfulness problem in medical AI, analyzing causes, summarizing mitigation methods and evaluation metrics, discussing challenges and limitations, and outlooking future directions. Existing research on investigating the factuality problem in medical AI remains in the initial phase, and there are several significant challenges to data resources, backbone models, mitigation methods, and evaluation metrics in this research direction. It is clear more future research efforts should be conducted and there are significant opportunities for novel faithful medical AI research involving adapting LLMs, and prompt learning et al. We hope this review can inspire further research efforts in this direction, as well as serve as a guide for researchers and practitioners on the safe use of AI methods in realistic medical practice.

## Figures and Tables

**Table 1:** The reference of different exemplar generative medical tasks for evaluating the factual consistency of the generated output by AI systems. "Ground truth" means the expected output of the model used for training.

Task	Input	Ground truth	Output	Reference
Medical text summarization	Medical texts	Summary written by experts	Short summary	Input
Radiology report summarization	Radiology report	Impression written by radiologists	Impression	Impression written by radiologists
Medical text simplification	Medical texts	Simplified texts written by experts	Simplified texts	Input
Medical dialogue generation	Dialogue from patients	Response written by experts	Response	Dialogue history
Medical question answering	Medical question	Correct answer written by experts	Answering	Correct answer written by experts
Radiology report generation	Chest X-ray image	Radiology report written by radiologists	Radiology report	Radiology report written by radiologists

**Table 2:** Methods for improving factuality of LLMs in medical.

Paper	Model	Research Method	Evaluation Task	Key Results
Yuan et al^79^	KeBioLM	Improve biomedical PLMs with UMLS	Biomedical NER, RE	Improve the performance of multiple biomedical PLMs such as BioBERT and PubMedBERT
Jha et al^80^	Biobert-continual	Integrate multiple knowledge bases into biomedical PLMs	Biomedical NER, RE, QA	Improve the performance of multiple biomedical PLMs such as BioBERT and PubMedBERT
Singhal et al^25^	Med-PaLM	Align a 540B LLM PaLM^78^ into the medical domain by instruction prompt tuning	Medical QA	Reduced factual errors in PaLM
Zakka et al^27^	Almanac	Augment LLMs with knowledge retrieval	Medical QA	Superior to ChatGPT on answering 20 medical questions
Nori et al^28^	GPT-4	Comprehensive study on GPT-4	Medical QA	Highlighted GPT-4's potential and limitations, such as factual inconsistency, and biases

**Table 3:** Methods for improving the factuality of medical AI methods for different generation tasks.

Paper	Task	Method for Improving Factuality	Evaluation Metrics
Zhang et al^17^	Summarization	RL	CheXpert F1, ROUGE
Delbrouck et al^35^	Summarization	RL with RadGraph reward	RadGraph, CheXpert F1, ROUGE
Xie et al^37^	Summarization	Rerank with RadGraph	RadGraph, CheXpert F1, ROUGE
Wallace et al^40^	Summarization	decoration and sorting strategy	ROUGE, DEI
Alambo^41^	Summarization	Incorporate medical NER and facts	ROUGE, UMLS F1, BioBERTScore
Yadav et al^42^	Summarization	RL maximizes medical question accuracy	ROUGE
Chintagunta et al^43^	Summarization	GPT-3 enhances data labeling	ROUGE, Concept F1, negation F1
Liu et al^44^	Summarization	medical knowledge retrieval and reasoning	ROUGE, METEOR
Lu et al^52^	Simplification	prompts enhance key phrase retention	ROUGE, BERTScore, BLEURT
Jeblick et at^54^	Simplification	ChatGPT	Human Evaluation
Lyu et al^55^	Simplification	ChatGPT	Human Evaluation
Nishino et al^58^	Generation	RL with clinical reconstruction score	ROUGE, CRS
Miura^59^	Generation	RL with entity matching	ROUGE, CheXpert F1, fact_ENT_
Delbrouck et al^60^	Generation	RL with RadGraph reward	CheXpert F1, fact_ENT_, RadGraph
Nishino et al^61^	Generation	RL with coordinated planning	ROUGE, ContentOrdering

**Table 4:** Datasets and methods for medical fact checking.

Paper	Datasets	Method for Fact Checking
Kotonya et al^65^	PUBHEALTH	BERT-based classifier and BERTSum-based summarizer
Wadden et al^66^	SCI-FACT	BERT-based methods
Wadden et al^74^	Red-HOT	Longerformer encoder with multi-task learning

## Data Availability

The datasets are available from authors upon reasonable request.
